# An expectation-maximization algorithm enables accurate ecological modeling using longitudinal microbiome sequencing data

**DOI:** 10.1186/s40168-019-0729-z

**Published:** 2019-08-22

**Authors:** Chenhao Li, Kern Rei Chng, Junmei Samantha Kwah, Tamar V. Av-Shalom, Lisa Tucker-Kellogg, Niranjan Nagarajan

**Affiliations:** 1 Computational and Systems Biology, Genome Institute of Singapore, Singapore, 138672 Singapore; 2 School of Computing, National University of Singapore, Singapore, 117543 Singapore; 3 Department of Microbiology and Immunology, University of British Columbia, Vancouver, V6T 1Z3 Canada; 4 Department of Computer Science, University of British Columbia, Vancouver, V6T 1Z4 Canada; 5 Centre for Computational Biology, Duke–NUS Graduate Medical School, Singapore, 169857 Singapore; 6 Yong Loo Lin School of Medicine, National University of Singapore, Singapore, 119228 Singapore

## Abstract

**Background:**

The dynamics of microbial communities is driven by a range of interactions from symbiosis to predator-prey relationships, the majority of which are poorly understood. With the increasing availability of high-throughput microbiome taxonomic profiling data, it is now conceivable to directly learn the ecological models that explicitly define microbial interactions and explain community dynamics. The applicability of these approaches is severely limited by the lack of accurate absolute cell density measurements (biomass).

**Methods:**

We present a new computational approach that resolves this key limitation in the inference of generalized Lotka-Volterra models (gLVMs) by coupling biomass estimation and model inference with an expectation-maximization algorithm (BEEM).

**Results:**

BEEM outperforms the state-of-the-art methods for inferring gLVMs, while simultaneously eliminating the need for additional experimental biomass data as input. BEEM’s application to previously inaccessible public datasets (due to the lack of biomass data) allowed us to construct ecological models of microbial communities in the human gut on a per-individual basis, revealing personalized dynamics and keystone species.

**Conclusions:**

BEEM addresses a key bottleneck in “systems analysis” of microbiomes by enabling accurate inference of ecological models from high throughput sequencing data without the need for experimental biomass measurements.

**Electronic supplementary material:**

The online version of this article (10.1186/s40168-019-0729-z) contains supplementary material, which is available to authorized users.

## Introduction

A growing body of literature points to the important roles that different microbial communities play in diverse natural environments [1, 2] and the human body [3]. This has particularly been aided by advances in next-generation sequencing technology, allowing for rapid, cost-effective taxonomic and functional profiling, combined with a computational analysis that has helped associate the state of the microbiome with various environmental conditions [1, 4] and human diseases [5–8]. Microbiomes are also constantly evolving, and there is now a growing appreciation that complex interactions between community members [9, 10] shape community dynamics [11, 12] as well as overall function [13, 14]. A systems view of the microbiome is thus essential for understanding and rationally manipulating it [15].

Because of its importance, there have been many approaches proposed to study microbial interactions and dynamics. Experimental approaches have ranged from simple two-species co-culture experiments [16–18] all the way to complex, multi-stage reactor models [19]. Analytical approaches [20] frequently use simple correlations between the abundances of various taxa in cross-sectional datasets to infer microbial interactions [21–23]. There are several challenges that need to be addressed in such analyses including the compositionality of sequencing data [21–24], low sensitivity and specificity of such methods [25, 26], and the inability to infer directionality of interactions or dynamics of the system [20].

The most commonly used approach for modeling microbial ecology is based on classical predator-prey systems, also referred to as generalized Lotka-Volterra models (gLVMs). gLVMs are based on ordinary differential equations (ODE) that model the logistic growth of species; naturally capture predator-prey, amensalistic, and competitive interactions; and have been applied to study dynamics of microbial ecosystems ranging from simple communities on cheese [27, 28] to the human microbiome [15, 26, 29–32]. More importantly, from a practical perspective, gLVMs have been used for a range of applications including identifying potential probiotics against pathogens [15, 29, 30], forecasting changes in microbial density, characterizing important community members (e.g., keystone species [26]), and analyzing community stability [30, 32, 33].

Despite this, a key limitation of gLVMs that restricts applicability and wider use is the requirement for microbial abundance data on an absolute scale. Microbiome analysis using high-throughput sequencing naturally provides relative abundance estimates with what is often referred to as “compositionality bias” [21, 22, 24] and cannot be directly used to estimate gLVM parameters [31]. Scaling relative abundances to an absolute scale typically requires additional experimental data that is either not readily available (as is true for the vast proportion of publicly available datasets), is technically challenging to directly quantitate for different sample matrices and complex communities (e.g., using flow cytometry [34, 35]), or can suffer from significant technical [36–38] and biological noise [39] (e.g., using 16S rRNA qPCR [15, 29, 30]).

In the face of these technical challenges, gLVM inference can seem daunting, especially because relative abundances do not seem to carry any information related to an absolute scale. Notably, we show that suitable scaling factors can be directly inferred from microbiome sequencing data, through an algorithm that couples biomass estimation and gLVM inference in an expectation-maximization (BEEM) [40] framework. This approach alternates between learning scaling factors and gLVM parameters and thus obviates the need for experimental scaling factors which otherwise limits the use of many existing datasets. Based on synthetic data where absolute cell density (biomass) is precisely known, we show that BEEM-estimated gLVM parameters are as accurate as those estimated with noise-free biomass values, and significantly more accurate than what could be expected with commonly used (16S rRNA-based) experimentally determined biomass estimates. Using data from a freshwater microbial community with flow cytometry-based gold-standard cell counts, we show that biomass estimated using BEEM has good concordance with the gold standard and improves significantly over the existing techniques to normalize data. Leveraging BEEM’s unique ability to learn gLVMs from relative abundance data, we analyzed publicly available datasets that represent the longest human gut microbiome time series data available to date [41–43]. This analysis highlighted the personalized dynamics of gut microbial biomass in different individuals, with communities driven by distinct interaction networks and hub species. Our analysis suggests an emergent model for gut microbial dynamics where relatively low abundance species may play key roles in maintaining gut homeostasis.

## Results

### Experimentally obtained biomass estimates can lead to inaccurate gLVMs

The gLV equations model the growth rate ($\frac{d{x}_i(t)}{dt}$) of each microbial species *i* as a function of absolute cell densities (*x*_*i*_(*t*)) of all the *p* species in a community:

1 $$ \frac{d{x}_i(t)}{dt}={\mu}_i{x}_i(t)+\sum \limits_{j=1}^p{\beta}_{ij}{x}_i(t){x}_j(t). $$

In the above model, the intrinsic growth rate parameter (*μ*_*i*_) and self-interaction parameters (*β*_*ii*_) define the logistic growth behavior of species *i*. In addition, the model also captures the impact of the absolute density of species *j* on the growth rate of species *i* through additional parameters (*β*_*ij*_, *i* ≠ *j*), assuming a linear and additive effects model. As high-throughput sequencing-based approaches to analyze microbiomes only provide relative abundance estimates, scaling factors related to the total biomass for each sample are then needed to accurately fit gLVMs in practice.

**Fig. 1:**
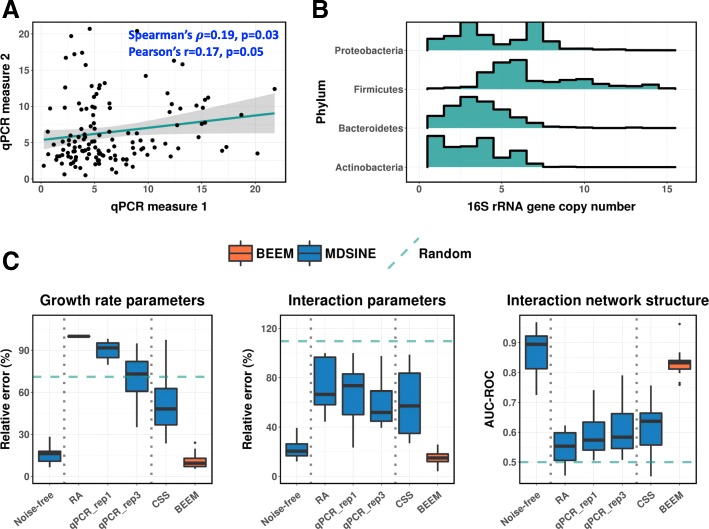
Noise in experimentally determined biomass severely distorts gLVM parameter estimation. **a** Scatter plot with fitted linear regression line for 2 16S qPCR technical replicates from Bucci et al. **b** Copy number variation for 16S rRNA genes in members of 4 major phyla of human gut bacteria. **c** Relative impact of different experimental (qPCR_rep1, 1 qPCR technical replicate; qPCR_rep3, mean of 3 qPCR technical replicates) and computational (RA, relative abundance; CSS, CSS normalization) data scaling approaches on gLVM parameter estimation (BVS algorithm for MDSINE), in comparison with using noise-free biomass or using BEEM. Boxplots represent the summary of 15 simulations (10 species, 30 replicates with 30 time points each), and 3 different metrics are shown here including median relative error for growth rate (***μ***) and interaction (***β***) parameters, and AUC-ROC for the interaction network. Dashed horizontal lines represent the performance of randomly generated parameters from the simulation model

The predominantly used approach to estimate total biomass is to quantify the copy number of the 16S rRNA gene using quantitative PCR (qPCR) [15, 29, 30]. However, 16S qPCR estimates have been reported to have a high technical noise, with a coefficient of variation (CV) ranging from 11 to 75% [36–38]. To reconfirm this, we reanalyzed 16S qPCR data from a recent microbiome modeling study on *Clostridioides difficile* infections [30] and observed low concordance across technical replicates (Spearman *ρ* < 0.22; Fig. 1a and Additional file 1: Figure S1A), as well as high coefficient of variation (mean CV = 51%). Another critical source of error with 16S qPCR-based biomass estimates is biological and arises due to the fact that bacteria can have a widely varying number of copies of the 16S rRNA gene, even within the same ecological niche. For example, the 16S gene copy number of the 4 major gut bacterial phyla cover a broad spectrum (Fig. 1b), ranging from a single copy to 15 copies [39]. Correspondingly, 16S qPCR-estimated biomass of a community dominated by *Firmicutes* can be twice as much as that of a community dominated by *Bacteroidetes*, even if both communities have exactly the same cell density (100% relative error). Such large relative errors can then have a significant impact on the accuracy of gLVMs estimated from the data, as we show below.

To test the impact of biomass estimation errors on model inference, we generated synthetic datasets (10 species community) based on the parameters inferred from real datasets, similar to the approach in Bucci et al. [29] (see the “Materials and methods” section). This framework allows us to carefully evaluate the impact of different levels of noise in a setting where model parameters are known. We noted that, given error-free biomass data, a state-of-the-art method (MDSINE [29]) was able to infer model parameters with median relative error < 20% and with ~ 90% median AUC-ROC (area under the sensitive-specificity tradeoff curve) for interaction terms (***β***; Fig. 1c, noise-free). However, as expected [31], directly using relative abundance estimates without scaling them increased the median relative error for parameter estimates to > 60% (Fig. 1c, RA), with AUC-ROC for interaction terms being comparable to the randomly generated parameters from the prior model for the simulation (Fig. 1c, random). Similar performance was obtained using another model fitting algorithm that works with relative abundance data and assumes small fluctuations in biomass values (LIMITS [26, 44]; Additional file 1: Figure S1B). Using simulated biomass data with error profile similar to real qPCR data (CV = 51%; without systematic errors due to varying copy number of the 16S rRNA gene; see the “Materials and methods” section), surprisingly, did not improve the performance substantially when one technical replicate was provided (Fig. 1c, qPCR_rep1), and even with three technical replicates, the growth rate parameter estimates (median relative error > 70%) were comparable to random (Fig. 1c, qPCR_rep3). These results highlight that experimental errors in biomass estimates can significantly impact the gLVM parameter estimation even in a relatively well-controlled setting where model assumptions are strictly applied.

### Joint estimation of biomass and model parameters with BEEM

In order to address the challenges of noisy experimental biomass data and, in general, to make the gLVM modeling more widely applicable where biomass estimates are not available, we explored the idea of learning gLVM parameters directly from relative abundance data. To achieve this, we first note that model Eq. 1 can be expressed in terms of relative growth rates by dividing both sides of the equation by *x*_*i*_(*t*):

$$ \frac{d{x}_i(t)}{dt}/{x}_i(t)=\frac{d\ln {x}_i(t)}{dt}={\mu}_i+\sum \limits_{j=1}^p{\beta}_{ij}{x}_j(t). $$

By explicitly introducing relative abundances ($\tilde{x}_{i}(t)$) and total biomass (*m*(*t*), where ${x}_i(t)=m(t)\tilde{x}_{i}(t)$), we get:

$$ \frac{d\left(\ln m(t)+\ln \tilde{x}_{i}(t)\right)}{dt}={\mu}_i+m(t)\sum \limits_{j=1}^p{\beta}_{ij}\tilde{x}_{j}(t). $$

The biomass terms on the left-hand side (LHS) of the equation can be eliminated by subtracting the equation of a selected species *r* from the equations for all other species, resulting in a new system:

$$ \frac{d{y}_i(t)}{dt}={a}_i+m(t)\sum \limits_{j=1}^p{b}_{ij}\tilde{x}_{j}(t),i\ne r, $$

where ${y}_i(t)=\ln \left(\tilde{x}_{i}(t)/\tilde{x}_{r}(t)\right)$ and the equations are re-parameterized by *a*_*i*_ and *b*_*ij*_, which are related to the original parameters (*a*_*i*_ = *μ*_*i*_ − *μ*_*r*_ and *b*_*ij*_ = *β*_*ij*_ − *β*_*rj*_). This new system has the advantage that all unknowns are on the right-hand side (RHS) of the equation and the gradient term on the LHS can be estimated directly from relative abundance data through spline smoothing and numerical differentiation [15, 26, 29, 30].

We then made the observation that the above equations can be re-written as two regression problems across two dimensions of the data matrix ($\tilde{x}_{i}(t),\forall i,t$):

- For each time point *t*, the biomass can be solved for via regression given the model parameters ***a*** and ***b*** for all the species.
- For each species *i*, the corresponding parameters *a*_*i*_ and *b*_*ij*_ can be solved through gradient matching [15, 26, 29, 30], given the biomass at each time point *t* (*m*(*t*)).

The interlock of the above two problems provides the basis for an expectation-maximization algorithm that alternates between estimating model parameters and biomass iteratively and forms the core of BEEM (see the “Materials and methods” section for details). Note that the estimates provided by BEEM for the biomass act as scaling factors to bring abundances across species and time points to the same scale for learning gLVMs.

On the synthetic datasets used in the previous section, we noted that despite not having any biomass data to work with, BEEM was a significant improvement over naïve analysis based on relative abundance data, as well as the results based on scaled relative abundances with noisy biomass data (~ 3× reduction in relative error; Fig. 1c, BEEM). In fact, BEEM-estimated parameters were nearly as accurate as those obtained using noise-free biomass data (relative error for growth rate and interaction terms), except for a slight decrease in AUC-ROC for interaction terms (primarily due to the rounding errors that provide non-zero estimates for zero terms). In comparison, other competing approaches (RA, qPCR, CSS) provided AUC-ROC performance similar to what is expected at random. Normalization approaches such as CSS [45] and TMM [46] (Fig. 1c, CSS; Additional file 1: Figure S1B; see the “Materials and methods” section) were tested here as control analytical methods but are not expected to work in general as they are designed to identify scaling factors that do not change across samples. We noted that BEEM’s significant improvement over other experimental and computational approaches and its ability to closely approximate analysis using noise-free biomass estimates is a robust feature that remains valid even when experimental biomass estimates are significantly better (CV = 5%, as expected from flow cytometry data) and while using different parameter estimation approaches or evaluation metrics (Additional file 1: Figures S1B and Figure S2).

### BEEM accurately estimates gLVM parameters and biomass in diverse model settings

**Fig. 2:**
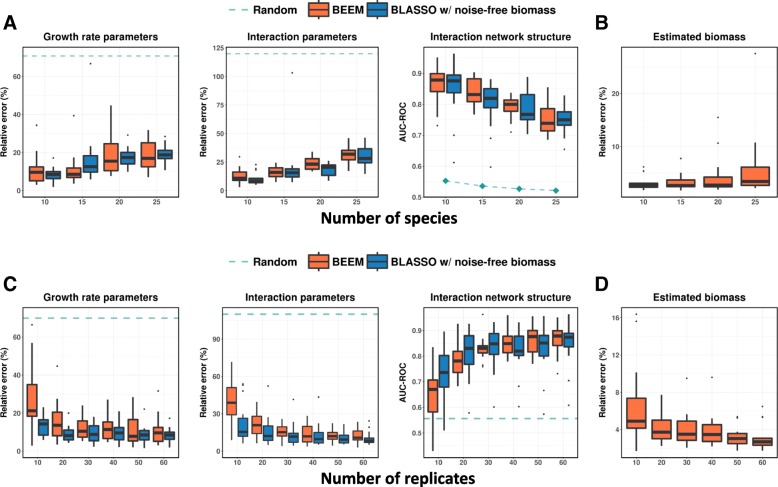
Robustness of parameter estimation with BEEM. **a** Results with an increasing number of species but fixed number of replicates (60). As expected, parameter estimation gets harder, but BEEM’s performance tracks the ideal case using BLASSO with noise-free biomass values, especially for interaction parameters. **b** Median relative error in biomass estimates remains less than 10%. **c** Results with an increasing number of replicates and fixed number of species (10). BEEM’s performance converges to that of BLASSO with noise-free biomass as the number of replicates increases. **d** Median relative error in biomass estimates reduces noticeably as the number of replicates increases

As in any situation where parameters have to be estimated, a sufficient number of data points (multiple biological replicates, referred to as replicates in the following sections) covering the dynamics of abundance change (e.g., recovery of the microbiome after a perturbation) are needed to get accurate gLVM models, and this in turn impacts the BEEM’s biomass estimates. In order to further study the BEEM’s performance characteristics, we generated synthetic datasets with a varying number of species and data points, comparing BEEM’s results to those obtained with noise-free biomass data and the same gradient matching algorithm (BLASSO, see the “Materials and methods” section) as used internally in BEEM. As expected, when the number of species increases but the number of data points remains constant (60 replicates with 30 time points), gLVM parameter estimation becomes harder (Fig. 2a). However, despite the quadratic increase in the number of parameters, the performance for both BLASSO (with noise-free biomass) and BEEM seems to only degrade linearly (Fig. 2a). In addition, even when the model has 25 species (650 model parameters) and can thus capture over 90% of the overall species abundance in a majority of human gut microbiomes [47] (but not all; Additional file 1: Figure S3), interaction parameters estimated by BEEM were nearly as accurate as those with noise-free biomass (Fig. 2a), though the growth rate parameters were more affected. For learning models with more species, a linear increase in the number of samples available was sufficient (Additional file 1: Figure S4). We also noted that the median relative error for biomass estimates from BEEM was generally well-controlled (< 10%; Fig. 2b).

Increasing the number of data points available for model fitting for a fixed number of species (10) improved the performance for both BLASSO with noise-free biomass and BEEM, as expected. Performance improvements were most notable when going from 10 to 20 replicates and plateaued out after that (30 time points; Fig. 2c). In general, after 20 replicates, differences between BLASSO and BEEM were small, especially in terms of estimating interaction parameters. Similarly, biomass estimates from BEEM had a median relative error < 5% when 20 replicates were available (Fig. 2d). In general, our analysis suggests that inherent limitations in gradient matching based on estimated gradients from data were a greater source of error for gLVM parameter estimation in many of our experiments than errors in BEEM-estimated biomass values. We also noted that some simulated datasets had significantly lower performance even when noise-free biomass values were provided, due to the presence of many time points that were close to equilibrium. Time points close to the equilibrium lead to noisy gradient estimates, and BEEM identifies and excludes such data points from its analysis (see the “Materials and methods” section).

**Fig. 3:**
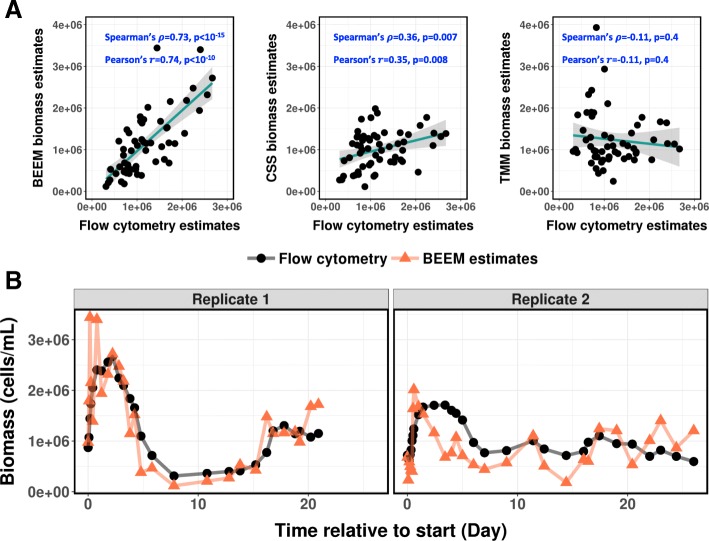
Concordance of BEEM-estimated biomass with gold-standard experimental measurements. **a** Scatter plots with fitted linear regression line highlighting that BEEM’s biomass estimates are notably more concordant with flow cytometry-based values compared to CSS and TMM normalization based estimates. **b** BEEM-estimated biomass values (orange) compared to gold-standard measurements using flow cytometry (black)

To assess BEEM’s performance for biomass inference in real-world datasets, we analyzed data from a recently published study on freshwater microbial communities [34, 35], which to our knowledge is the only one to have longitudinal microbiome sequencing data as well as flow cytometry-based gold-standard biomass estimation. Notably, the flow cytometry data in this study was reported to have high reproducibility (CV < 5%) [34] and therefore was suitable for use as the ground truth for total biomass. Surprisingly, with only 57 time points in total across 2 replicate experiments, BEEM was able to infer the total biomass for a 26-species community accurately solely based on relative abundances from 16S sequencing. BEEM-estimated biomass values showed a strong correlation with flow cytometry data (BEEM: Spearman’s *ρ* = 0.73, Pearson’s *r* = 0.74; Fig. 3a), and its trajectories closely tracked measured fluctuations (Fig. 3b). In contrast and as expected, normalization approaches provided estimates that had either weak correlation (CSS: Spearman’s *ρ* = 0.36, Pearson’s *r* = 0.35) or negative correlation with experimentally determined values (TMM: Spearman’s *ρ* = − 0.11, Pearson’s *r* = − 0.11; Fig. 3a).

Considering the lack of data for real microbial communities with well-characterized interactions, we pooled experimentally the measured growth characteristics for seven different species in a dense time series and used the data to compute the relative abundances for a community over time that would evolve under the assumption of no inter-species interactions. In addition to accurately estimating the biomass (Additional file 1: Figure S5A), BEEM was found to have a low false-positive rate (< 3% and < 8% using the most stringent and default thresholds, respectively) out of a total of 42 possible interaction terms (Additional file 1: Figure S5B). MDSINE, however, had much higher false-positive rates using different scaling approaches including with the true biomass values (> 26% and > 52% using the most stringent and default thresholds, respectively).

### Personalized gut microbial dynamics and keystone species

The development of BEEM allows us to analyze previously generated datasets in a gLVM framework, even when biomass measurements were not made in the original study. To showcase this capability, we applied BEEM to the longest (over 1 year) and most densely (almost daily) sampled human gut microbiome time series datasets available to date (four individuals: DA, DB from David et al. [42] and M3, F4 from Caporaso et al. [41]; individually modeled assuming sufficient perturbations to reveal dynamics). BEEM-estimated models exhibited a good fit to the data, with predicted relative abundances for a day based on numerical integration from the previous day being in high concordance with the observed data (median Spearman’s *ρ* = 0.84, median Pearson’s *r* = 0.90). In addition, BEEM-inferred growth rates were found to be concordant with the growth rates reported in the AGORA database based on the genome-scale metabolic modeling (Spearman’s *ρ* = 0.79, Pearson’s *r* = 0.74; Additional file 1: Figure S6) [48]. Finally, BEEM correctly identified several key interactions that have previously been validated using low-throughput experiments, including the inhibitory interactions between *Bacteroides uniformis* and *Enterobacteriaceae* [49], *Feacalibacterium prausnitzii* and *Enterobacteriaceae* [50–52], and *B. uniformis* and *F. prausnitzii* [53].

**Fig. 4:**
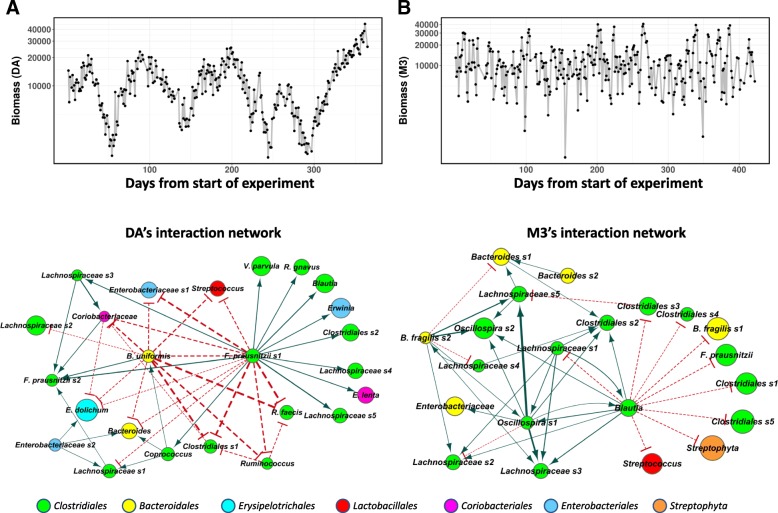
BEEM analysis of year-long gut microbial time series datasets. **a**, **b** BEEM-estimated biomass values for two individuals (DA and M3) with daily sampled, year-long gut microbial time series datasets from David et al. [42] and Caporaso et al. [41]. Interestingly, while M3’s biomass fluctuates rapidly, DA’s biomass seems to vary in a more defined fashion with a periodicity of around 3 months. **c**, **d** Graphs representing non-zero interaction terms in gLVM models learnt individually for DA and M3 using BEEM. Dashed and solid edges represent positive and negative interactions, respectively. Edge widths are proportional to the interaction strength, and node sizes are proportional to the log-transformed mean relative abundance of the corresponding species. Nodes are labeled with the most specific taxonomic annotations and colored according to order level information

As BEEM directly infers daily biomass values, we plotted these and observed distinct individual-specific patterns: while subject DA’s biomass was found to vary relatively smoothly, following an approximately cyclic pattern with a period of about 3 months (Fig. 4a), subject M3’s biomass fluctuated to a greater extent on a day-to-day basis with no clear trend (Fig. 4b). Similar patterns were observed in parts for subjects DB and F4, which had a greater resemblance to DA overall (Additional file 1: Figure S7A, B). The fluctuations predicted in M3’s biomass were also found to be present alongside (but not correlated with, *ρ* < 0.14) frequent blooms of rare taxa (relative abundance) that were not detected at other time points [43] and maybe a consequence of this instability in the community. In contrast, the smoother progression of DA’s biomass may be a reflection of the relative stability of the gut community in this individual, though the source of the observed cyclic patterns deserves to be explored further. As an initial hint, we noted that the strongest association between DA’s biomass and reported metadata was a negative correlation with calcium intake (Additional file 1: Figure S8).

We visualized the interaction terms predicted by BEEM as a weighted directed network for each individual (Fig. 4c, d; Additional file 1: Figure S7C, D). Concordant with their distinct biomass dynamics, DA and M3 also exhibited microbial interaction networks that were unique to them (Fig. 4c, d). DA’s network was defined by hub nodes for *Feacalibacterium prausnitzii* (s1) and *Bacteroides uniformis*, two species with many beneficial roles and frequent associations with a healthy gut [54, 55]. The hubs were found to negatively affect the growth of an *Enterobacteriaceae* species (s1), consistent with previous reports for *B. uniformis* [49] and *F. prausnitzii* [50–52]. In comparison, the major hub nodes in M3’s network were a *Blautia* and an *Oscillospira* species (s1) that were connected by a positive feed-forward loop. Additionally, we found that abundances of the *Blautia* and *Oscillospira* species were significantly negatively correlated with total biomass in M3’s gut microbiome (Additional file 1: Figure S9). Feed-forward loops have been implicated in destabilizing effects on ecosystems [32], and so these observations may explain the unstable behavior of M3’s biomass as well as the corresponding susceptibility to invasive blooms of rare taxa [43]. *Blautia*’s protective role in M3’s gut flora is further indicated by its inhibition of *B. fragilis* (s1), an opportunistic pathogen that has been associated with diarrhea [56]. Interestingly, several of the transient species in M3’s gut microbiome were observed to be at the periphery of the network, with a single incoming edge indicating that their abundances were being influenced by a hub species. For example, this was observed for several *Streptococcus* species that are primarily oral commensals and could be transient colonizers of the gut [57, 58].

Despite the differences in the identity of species in their interaction networks, the various individual-specific networks shared some common features, including the presence of a few hub nodes that negatively influenced many other species, and were generally not the most abundant species in the community (Fig. 4c, d; Additional file 1: Figure S7C, D). Overall, we also found that the ratio between out- and in-degree of species in the networks was negatively correlated with their mean relative abundances (Additional file 1: Figure S10), suggesting that the hub species in the interaction network, which are often considered as keystone species for the community [26, 59], are typically not the abundant species in the gut microbiome. We further confirmed this observation by analyzing a large collection (840 healthy individuals) of gut microbiome datasets [47], to find that the core species in the gut microbiome were also frequently not the most abundant species (Additional file 1: Figure S11). Together, these observations suggest a model for the gut microbiome where relatively less abundant species in the community are more stable colonizers of the host, and by virtue of their impact on the growth of other species in the community, play an important role in defining its dynamics in different individuals.

## Discussion

A major limitation of most microbiome profiling datasets available to date is the restriction to relative abundances and the “compositionality” of this data has led to significant challenges even when performing common statistical tests for correlated abundances [60]. These issues are amplified when considering systems models such as gLVMs, and our analysis here confirms that the model parameter estimates can be severely distorted if relative abundances are not correctly scaled. In ecological models such as gLVMs, interactions between species are naturally a function of the absolute density of species in a community rather than their relative abundances [61, 62]. Correspondingly, while autoregression-based methods such as sVar [43] and ARIMA [63] provide an alternative for model fitting with relative abundance data, ecological interpretations for their models and parameters have not been put forward (e.g., species growth rate or carrying capacity). In addition, experimental approaches to measure scaling factors are generally seen as a laborious and occasionally feasible way to work with absolute abundances. However, as we show here, this may not be the case if care is not taken to ensure that experimental noise is minimized and a sufficient number of technical replicates are analyzed. By eliminating the need for additional experimental data, BEEM greatly expands the applicability of gLVMs to the microbiome datasets, and its robustness could simultaneously improve the quality of models and scaling factor estimates, as observed in our synthetic and real datasets. Explicitly modeling microbial interactions through gLVMs has proven to be a powerful framework for studying microbial community dynamics [15, 26–32], and the approach used in BEEM could also be extended (with minimal modifications) to time series with external perturbations (e.g., antibiotics usage) [15, 29, 30], as well as system models for gene expression regulation based on RNA-seq data [64].

Due to limited availability of absolute abundance data, gLVMs have generally been constructed by aggregating information across experiments and individuals [15, 29, 30]. We exploited the availability of year-long time series datasets and BEEM’s facility with relative abundances to construct individual specific gut microbiome gLVMs. Intriguingly, we observed that our inferred scaling factors suggest that gut microbial biomass has distinct dynamics across different individuals. Consistent with a recent study on 20 individuals where human gut microbial biomass (measured via flow cytometry) was found to have high variation (CV ≈ 53% within a week) [60]; we also noted high variability over time across the four individuals we analyzed (CV ranging from 49 to 76% over a year). Misleading conclusions are likely to be drawn without accounting for such variation, and BEEM-estimated biomass values may be useful with other statistical and ecological modeling methods as well. Additionally, we observed cyclic behavior of biomass trajectories in multiple individuals, similar to the seasonal patterns reported in hunter-gatherers of Western Tanzania [65], and the conserved patterns observed in other mammals across evolutionary timescales [66]. Similar patterns have not been reported before for western city dwellers, perhaps due to the confounding effects of aggregate analysis across individuals and the impact of highly diverse diets. BEEM analysis, however, suggests that the underlying patterns may still be conserved in urban subjects and may be more general than previously believed.

Our inference of the gLVM models for each individual allows us to identify specific microbial species and the kinds of interactions that they have, to account for the distinct dynamics that were observed. For example, the positive feed-forward loop observed between the hubs in M3’s gut microbiome provides a specific, plausible, and testable hypothesis to explain the instability observed there, and this capability can be valuable in future studies where targeted interventions are feasible. Despite the differences in the microbial interaction networks observed for different individuals, a shared feature seems to be the presence of relatively lowly abundant species that act as hub nodes in the network. A similar pattern was seen in cross-sectional data as well where frequently shared “core” gut microbiome species tend to not be the most abundant species in the community. These observations point to a model where species at low relative abundances stably colonize the gut (e.g., mucosa-associated ones) compared to abundant but transient (lumen-associated) bacteria and play an important role in defining gut microbiome dynamics. In particular, hub species were frequently found to negatively regulate more transient species in the community, in agreement with the known role of mucosa-associated species in providing colonization resistance against invasive pathogenic species [67]. We envisage that perturbation experiments with in vitro [68] and in vivo systems [69] could help further validate such predictions and the ability to forecast abundance changes using gLVMs learnt by BEEM.

An important point that we noted in the gut microbiome datasets that were analyzed here is the limited number of stable species (prevalent in most time points for an individual) that are shared across individuals. This feature makes it infeasible to learn gLVM models by merging short time series datasets across different individuals. Similar constraints might be present in other microbial communities as well, including specific challenges in measuring total biomass in complex matrices [60], and thus, the development of BEEM makes it more feasible to generate the long and densely sampled datasets that are needed for such models. As the complexity (number of species) of modeled communities grows, BEEM models also require a linear increase in the number of available samples. The analysis in BEEM can potentially be directly extended to cross-sectional datasets if the corresponding communities are believed to be at equilibrium (i.e., $\frac{d{x}_i(t)}{dt}=0$, for all species). This extension would significantly expand the amount of data that could be used and thus allow us to learn even more complex models in the future. As is the case for any modeling approach, no model is expected to be perfect, but as they capture more and more features of real systems, we can expect that their predictions become increasingly useful. BEEM’s development therefore serves as an important step in expanding the use of modeling approaches to study microbial community dynamics and rationally identify appropriate perturbations.

## Conclusions

We present a novel algorithm, BEEM, that addresses a key bottleneck in “systems analysis” of microbiomes by enabling accurate inference of ecological models from time course high-throughput microbiome sequencing data without the need for experimental biomass measurements. This approach circumvents the limitations of 16S rRNA qPCR-based biomass measurement and its underappreciated adverse impact on model fitting accuracy. BEEM’s robustness was established based on systematic evaluations with synthetic and real datasets. Its application to year-long human gut microbiome data revealed novel insights into personalized microbiome dynamics driven by distinct keystone species. We therefore expect BEEM to be a useful tool for the microbiome community in obtaining deeper insights into how microbial interactions determine system-level behavior.

## Materials and methods

### BEEM’s core algorithm

As introduced in the “Joint estimation of biomass and model parameters with BEEM” section, the gLVM model in Eq. 1 can be first simplified by dividing *x*_*i*_(*t*) on each side and then re-written in terms of total biomass *m*(*t*) (i.e., $m(t)={\sum}_{i=1}^p{x}_i(t)$) and relative abundances $\tilde{x}_{i}(t)$ (i.e., $\tilde{x}_{i}(t)={x}_i(t)/m(t)$) as shown below:

2 $$ \frac{d\ln m(t)+\ln \tilde{x}_{i}(t)}{dt}={\mu}_i+m(t)\sum \limits_{j=1}^p{\beta}_{ij}\tilde{x}_{j}(t). $$

To eliminate the biomass-related term in the LHS of the equation, we subtract the corresponding equation for a reference species *r* (species with lowest CV, by default) from both sides of the system, resulting in additive log ratio (ALR)-transformed [70] relative abundances (${y}_i(t)=\ln \left(\tilde{x}_{i}(t)/\tilde{x}_{r}(t)\right)$) on the LHS and a re-parameterized RHS:

$$ \frac{d{y}_i(t)}{dt}={a}_i+m(t)\sum \limits_{j=1}^p{b}_{ij}\tilde{x}_{j}(t),i\ne r, $$

where *a*_*i*_ = *μ*_*i*_ − *μ*_*r*_ and *b*_*ij*_ = *β*_*ij*_ − *β*_*rj*_.

An estimate for *dy*_*i*_(*t*)/*dt*, denoted as *Y*_*it*_, can be calculated as the derivative of a piece-wise polynomial spline fitted to the ALR-transformed relative abundances (*y*_*i*_(*t*), see the “Robust parameter estimation with BEEM” section for details). Given the following model for *p* species:

$$ {Y}_{it}={a}_i+{m}_t\sum \limits_{j=1}^p{b}_{ij}{\overset{\sim }{X}}_{jt}+\epsilon, \epsilon \sim \mathrm{Normal}\left(0,{\sigma}^2\right), $$

where ${\overset{\sim }{X}}_{it}=\tilde{x}_{i}(t)$ and *m*_*t*_ = *m*(*t*) are the variables written in their matrix representations, we can write the following likelihood function:

$$ \mathcal{Q}\left(\boldsymbol{a},\boldsymbol{b}|{\boldsymbol{a}}^{\left(T-1\right)},{\boldsymbol{b}}^{\left(T-1\right)}\right)={E}_{\boldsymbol{M}\mid {\boldsymbol{a}}^{\left(T-1\right)},{\boldsymbol{b}}^{\left(T-1\right)},\boldsymbol{X},\boldsymbol{Y}}\left[L\left(\boldsymbol{a},\boldsymbol{b};\boldsymbol{X},\boldsymbol{Y},\boldsymbol{M}\right)\right]=\int L\left(\boldsymbol{a},\boldsymbol{b};\boldsymbol{X},\boldsymbol{Y},\boldsymbol{M}\right)\delta \left(\boldsymbol{M}-\boldsymbol{m}\right)d\boldsymbol{M}=L\left(\boldsymbol{a},\boldsymbol{b};\boldsymbol{X},\boldsymbol{Y},\boldsymbol{m}\right)=\prod \limits_t\prod \limits_i\frac{1}{\sqrt{2\pi {\sigma}^2}}{e}^{-\frac{{\left({Y}_{it}-\left({a}_i+{m}_t\sum \limits_{j=1}^p{b}_{ij}{\overset{\sim }{X}}_{jt}\right)\right)}^2}{2{\sigma}^2}}, $$

where ***a*** and ***b*** are the model parameters, *δ*(***M***) is a Dirac delta function for the biomass values, and *L*(***a***, ***b***; ***X***, ***Y***, ***M***) is the likelihood function with respect to ***a*** and ***b*** for the above regression problem. The parameters are then solved with the following EM algorithm.

#### Biomass estimation (E-step)

In iteration *T*, with ${\hat{a}}_i^{\left(T-1\right)}$ and ${\hat{b}}_{ij}^{\left(T-1\right)}$ from the previous iteration, the biomass ${\hat{m}}_t^{(T)}$for each *T* can be computed as the coefficient of the following linear regression:

$$ {U}_{ti}^{(T)}\sim {m}_t^{(T)}{V}_{ti}^{(T)},i\ne r, $$

where ${U}_{ti}^{(T)}={Y}_{it}-{\hat{a}}_i^{(T)}$ and ${V}_{ti}^{(T)}=\sum \limits_{j=1}^p{\hat{b}}_{ij}^{(T)}{\overset{\sim }{X}}_{jt}$. Note that accurate estimation of biomass through this regression requires a sufficient number of data points (number of species > 6), and BEEM will warn users if this is not the case.

#### Model parameter estimation (M-step)

With estimated biomass from the E-step, ${\hat{\boldsymbol{m}}}^{(T)}$, BEEM estimates ${\hat{a}}_i^{(T)}$ and ${\hat{b}}_{ij}^{(T)}$ for each *i* (*i* ≠ *r*) based on the following regression problem (also known as gradient matching):

$$ <{\hat{\boldsymbol{a}}}^{(T)},{\hat{\boldsymbol{b}}}^{(T)}>=\underset{\boldsymbol{a},\boldsymbol{b}}{\mathrm{argmax}}\log \left(\mathcal{Q}\left(\boldsymbol{a},\boldsymbol{b}|{\boldsymbol{a}}^{\left(T-1\right)},{\boldsymbol{b}}^{\left(T-1\right)}\right)\right)=\underset{\boldsymbol{a},\boldsymbol{b}}{\mathrm{argmax}}\log \left(L\left(\boldsymbol{a},\boldsymbol{b};\boldsymbol{X},\boldsymbol{Y},{\hat{\boldsymbol{m}}}^{(T)}\right)\right). $$

Solving the above system is often limited by the amount of data available in practice. For microbial communities, it is usually assumed that the interaction vector (*β*_*ij*_) is sparse (i.e., a species is only directly affected by a small number of other species). Consequently, the transformed matrix *b*_*ij*_ is also sparse, and BEEM estimates it using a sparse regression technique based on a Bayesian approach (Bayesian lasso—BLASSO [30]; R package “monomvn” version 1.9-7; default parameters) [71].

#### Initialization

For the initialization step in its EM algorithm, BEEM assumes that scaling factors inferred from a commonly used normalization approach for microbiome data (cumulative sum scaling—CSS [45]) provides a reasonable starting point for the algorithm to then learn better scaling factors. Note that, as expected, scaling factors from CSS normalization and BEEM cannot recapitulate the absolute scale corresponding to experimental measurements (e.g., by qPCR or flow cytometry), and so their estimates were scaled to the same median value across the time series as experimental measurements for subsequent comparisons. In practice, the true scale of all samples can be recovered by measuring the biomass for a single sample accurately. BEEM implementation also checks to ensure that sufficient number of data points are available to estimate gLVM models for the given number of species (number of data points > number of parameters) and will warn users otherwise. Time points near equilibrium (> 80% species that change < 5% in relative abundance) are excluded from BEEM analysis to avoid noise in gradient estimation.

#### Termination and parameter estimation

The E- and M-steps in BEEM are run until convergence or a user-specified maximal number of iterations. The search was assumed to have reached convergence (to a local optimum) when the mean squared error (MSE, smoothed using a moving median with a window size of 3) for the E-step varies by less than a user-specified tolerance (0.1% by default) for 3 consecutive iterations [72]. In practice, on the real datasets analyzed in this study, convergence takes ~ 1 h using 4 CPUs. Estimates for ${\hat{a}}_i$, ${\hat{b}}_{ij}$, and ${\hat{m}}_t$ were calculated as the median of the values from all iterations (excluding the first 30 iterations) whose MSE was within 5% of the minimal MSE. BEEM throws a warning message if it does not converge within the user-specified number of iterations or if the observed fit to the data is poor (biomass-normalized MSE > 10^−5^).

### Robust parameter estimation with BEEM

In our experiments with synthetic and real data, we noted that gLVM modeling can be sensitive to noise and outliers in the data, and this in turn could affect estimation of scaling factors with BEEM. To address this, we refined the core algorithm in BEEM with additional pre-processing steps that further enable robust parameter estimation.

#### Outliers in relative abundance data

We observed in our numerical analysis that outliers in the abundance data could notably affect the spline fitting procedure and lead to spurious gradient estimates. To obtain more robust spline fitting, an over-smoothed spline was first fitted to *y*_*i*_(*t*) (function “smooth.Pspline” from R package “pspline” [73] with maximal degree of 5 and a large smoothing parameter “spar = 1e10”) to calculate the absolute error in fitted values (*e*_*it*_ =  ∣ *y*_*i*_(*t*) − *y*_*i*_(*t*)^smoothed^∣), and points with absolute error larger than expected ($\left({e}_{it}-\underset{j}{\mathrm{median}}\left({e}_{ij}\right)\right)/\underset{j}{\mathrm{MAD}}\left({e}_{ij}\right)>\tau$, *τ* = 5 by default) were then filtered out. The final smoothing spline was fitted (degree of 5 and smoothing parameter selected using cross validation) to the remaining data to calculate the estimated gradients *Y*_*it*_. In addition, outliers in biomass estimated from the previous iteration (${\hat{m}}_t^{\left(T-1\right)}$) were identified in the same way and replaced with interpolated values from the spline.

#### Outliers in estimated gradients

In practice, gradient matching-based methods (including the various algorithms implemented in MDSINE) were found to be sensitive to outliers in the estimated gradients (i.e., *Y*_*it*_). To identify outliers in a time series (*Y*_*it*_, for all *t*), a local regression (LOESS) smoother was fitted to de-trend *Y*_*it*_, and the outliers were filtered out as described above.

#### Estimating constrained biomass values

For each time point, biomass was estimated as the slope of a linear regression (${U}_{tk}^{(T)}$ against ${V}_{tk}^{(T)}$) where outliers in both ${U}_{tk}^{(T)}$ and ${V}_{tk}^{(T)}$ were identified and removed following a standard boxplot approach, i.e., as deviations from the median by more than 1.5× inter-quartile range. In addition, the biomass was constrained to be positive by removing points where ${U}_{ti}^{(T)}$ and ${V}_{ti}^{(T)}$ had different signs.

### Recovering gLVM parameters

Based on the previously stated assumption that the interaction matrix ***β*** is sparse, most entries in each column are expected to be zero and thus the median value for the *j*th column in ***b*** would be expected to be −*β*_*rj*_, allowing us to infer back all the other rows of ***β*** (*β*_*ij*_ = *b*_*ij*_ + *β*_*rj*_, default implementation in BEEM’s “paramFromEM” function). BEEM then assigns a *Z*-score like confidence value (*s*_*ij*_) to each entry of ***β***, by dividing the estimated interaction strength by the column standard deviation (${s}_{ij}=\mid {\hat{\beta}}_{ij}/{\sigma}_j\mid$). The growth rate vector ***μ*** is not expected to be sparse but can be recovered by directly solving the original gLVM system (Eq. 2), using the already derived estimates for scaling factors and ***β***. For robustness, BEEM estimates the growth rate for each species as the median of positive estimates across all time points. BEEM also provides a “non-sparse” mode (setting argument “sparse” to “FALSE” in the “paramFromEM” function) to estimate all parameters by solving the gLVM system directly with estimated biomass values.

### Datasets and evaluation metrics

#### Simulated datasets

MDSINE’s Bayesian variable selection (BVS) algorithm (with spline smoothing option and minor bug fixes: https://bitbucket.org/chenhao_li/mdsine) was used to estimate the parameters from the *C. difficile* infection dataset provided with the package [30]. Simulated datasets were then generated based on these estimated parameters following the procedure described in Bucci et al. [30] (excluding perturbations) by numerically integrating the gLVM with randomly generated initial states (mimicking the recovery of the microbiome after a random perturbation). Unless stated otherwise, we generated simulated data with 10 species, 60 replicates (with different random initial states) with 30 time points each. Noisy abundances were obtained by sampling from Poisson distributions [74] with means based on scaled abundances at each time point (sum = 5 × 10^4^). Simulated qPCR and flow cytometry-based values for total biomass were generated from log-normal distributions with coefficients of variation (CV) that matched those seen in real datasets (qPCR = 51% [30], flow cytometry = 5% [34, 35]). For each condition with varying number of species or replicates and different biomass estimation techniques, 15 simulated datasets with different model parameters were tested.

#### Dataset from Props et al.

The original OTU table was obtained from the authors [35]. Samples for the “operation” stage, where the environment had roughly constant temperature were selected for BEEM analysis. OTUs with low mean relative abundances (< 0.1%) were excluded to ensure that sufficient data is available to fit the model parameters, resulting in 26 OTUs across 58 time points from 2 replicates.

#### Dataset from Gibbons et al.

This dataset included 4 long time series collected by David et al. [42] and Caporaso et al. [41]. To reduce the number of OTUs to model and remove OTUs not detectable in many samples, the original OTU tables [43] were filtered to keep only top OTUs based on prevalence (> 10 reads in most of the samples). In total, 26 and 22 OTUs were left for samples from David et al. and Caporaso et al., respectively. In order to assess the robustness of the inferred network, BEEM was run with 30 different seeds, and edges with confidence score *s*_*ij*_ ≤ 1 in more than 50% of the networks were kept. The final biomass was obtained by taking the geometric mean across all 30 runs (Additional file 2).

#### Growth curve data

Seven different bacterial species were separately inoculated into triplicate wells of a Bioscreen honeycomb microplate containing brain heart infusion (BHI) broth. Absorbance values were measured at 600 nm (OD600) every 20 min for 48 h for the microplate incubated in Bioscreen C at 37 °C with continuous shaking at high amplitude and normal speed. The OD600 values for the lag and stationary phases were removed, resulting in 10 time points for each species (Additional file 3).

#### Metrics for evaluation

The following metrics were used for evaluating inference algorithms:

- Median relative error (MRE) for estimates $\hat{\boldsymbol{\theta}}$ when the true values are ***θ***: $\underset{\theta_i\ne 0}{\mathrm{median}}\left|\frac{{\hat{\theta}}_i-{\theta}_i}{\theta_i}\right|$.
- Area under the receiver operating characteristic curve (AUC-ROC) for the inferred microbial interactions. Confidence scores from BEEM were used to rank predicted interactions and to compute the AUC-ROC value.

#### MDSINE and LIMITS

The two algorithmic settings in MDSINE, BLASSO, and Bayesian variable selection (BVS) were both run with and without the spline fitting option (other parameters were kept at default values). LIMITS (implemented in the R package seqtime_0.1.1 [44]) was run with default parameters. To compute AUC-ROC values, Bayesian factors were used to rank the interactions for BVS, while the absolute values of parameters were used for BLASSO and LIMITS.

## Additional files

Additional file 1: **Supplementary Figure 1:** Noise in experimentally determined biomass severely distorts gLVM parameter estimation. **Supplementary Figure 2:** The impact of noise on the performance of different gLVM parameter estimation algorithms is similarly captured with other evaluation metrics as well. **Supplementary Figure 3:** Relative abundances observed for the most abundant species in 840 normal stool metagenomic samples from Pasolli et al. **Supplementary Figure 4:** Boxplots of relative error in BEEM estimated parameters from data with different number of species. Each box represents 30 independent simulations. **Supplementary Figure 5:** BEEM effectively controls for false positive interactions in a synthetic community with no interactions. **Supplementary Figure 6:** Scatter plot for predicted growth rates from BEEM versus growth rates reported in the AGORA database based on genome-scale. metabolic models. **Supplementary Figure 7:** BEEM estimated biomass and interaction networks from the two shorter gut microbial longitudinal profiles from David et al and Caporaso et al. **Supplementary Figure 8:** Changes in calcium intake for the preceding day in relation to BEEM-estimated biomass for subject DA’s gut microbiome. **Supplementary Figure 9:** Scatter plots with fitted linear regression lines between the two hub OTUs and the estimated biomass of M3’s gut microbiome. **Supplementary Figure 10:** Scatter plot with fitted linear regression line between the out- and in-degree of the OTU versus its mean relative abundance on log scale. **Supplementary Figure 11:** Core species of gut microbiome are often not among the top abundant species. (PDF 4735 kb)

Additional file 2: GLVM parameter estimates for the gut microbiomes of the four subjects. (XLSX 51 kb)

Additional file 3: OD600 measurements for 10 different species. (XLSX 10 kb)
