# Supplementary material for: An expectation-maximization algorithm enables accurate ecological modeling using longitudinal microbiome sequencing data
Source: Microbiome. 2019 Aug 22;7:118. doi: 10.1186/s40168-019-0729-z (PMC6706891; doi:10.1186/s40168-019-0729-z)
Supplement: Supplementary file 1 — Supplementary Figure 1: Noise in experimentally determined biomass severely distorts gLVM parameter estimation. Supplementary Figure 2: The impact of noise on the performance of different gLVM parameter estimation algorithms is similarly captured with other evaluation metrics as well. Supplementary Figure 3: Relative abundances observed for the most abundant species in 840 normal stool metagenomic samples from Pasolli et al. Supplementary Figure 4: Boxplots of relative error in BEEM estimated parameters from data with different number of species. Each box represents 30 independent simulations. Supplementary Figure 5: BEEM effectively controls for false positive interactions in a synthetic community with no interactions. Supplementary Figure 6: Scatter plot for predicted growth rates from BEEM versus growth rates reported in the AGORA database based on genome-scale. metabolic models. Supplementary Figure 7: BEEM estimated biomass and interaction networks from the two shorter gut microbial longitudinal profiles from David et al and Caporaso et al. Supplementary Figure 8: Changes in calcium intake for the preceding day in relation to BEEM-estimated biomass for subject DA’s gut microbiome. Supplementary Figure 9: Scatter plots with fitted linear regression lines between the two hub OTUs and the estimated biomass of M3’s gut microbiome. Supplementary Figure 10: Scatter plot with fitted linear regression line between the out- and in-degree of the OTU versus its mean relative abundance on log scale. Supplementary Figure 11: Core species of gut microbiome are often not among the top abundant species. (PDF 4735 kb) [file 40168_2019_729_MOESM1_ESM.pdf]

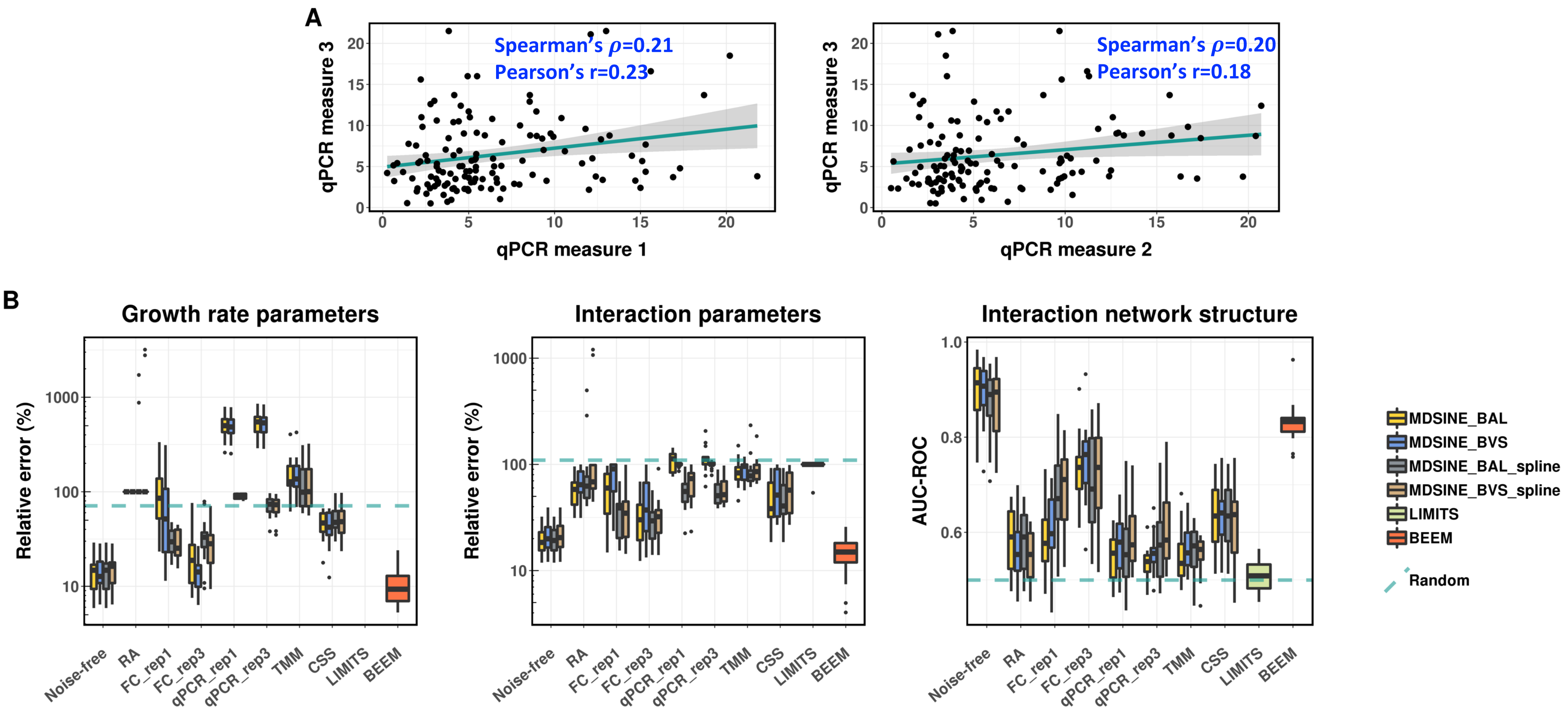

Supplementary Figure 1. Noise in experimentally determined biomass severely distorts gLVM parameter estimation. (A) Scatter plots with fitted linear regression lines for three 16S qPCR technical replicates from Bucci *et al* (Left: p-value=0.021 and 0.009 for Spearman and Pearson correlations; Right: p-value=0.014 and 0.046 for Spearman and Pearson correlations). (B) Relative impact of different gLVM parameter estimation algorithms (BAL, BVS, BAL_spline and BVS_spline as implemented in MDSINE) and data scaling approaches. Boxplots represent the summary of 15 simulations (10 species, 30 replicates with 30 time points each). Note that, in general, different data scaling approaches were found to impact performance more than the different estimation algorithms. Dashed horizontal lines represent the performance of randomly generated parameters from the simulation model. In general, scaling with noise-free biomass factors and using BEEM provided notably good results, and among competing experimental (FC_rep1, FC_rep3, qPCR_rep1, qPCR_rep3) and computational approaches (RA – relative abundance, CSS – CSS normalization, TMM – TMM normalization, LIMITS), having three replicates from flow cytometry was the closest (FC_rep3). Note that LIMITS does not compute growth rate parameters.

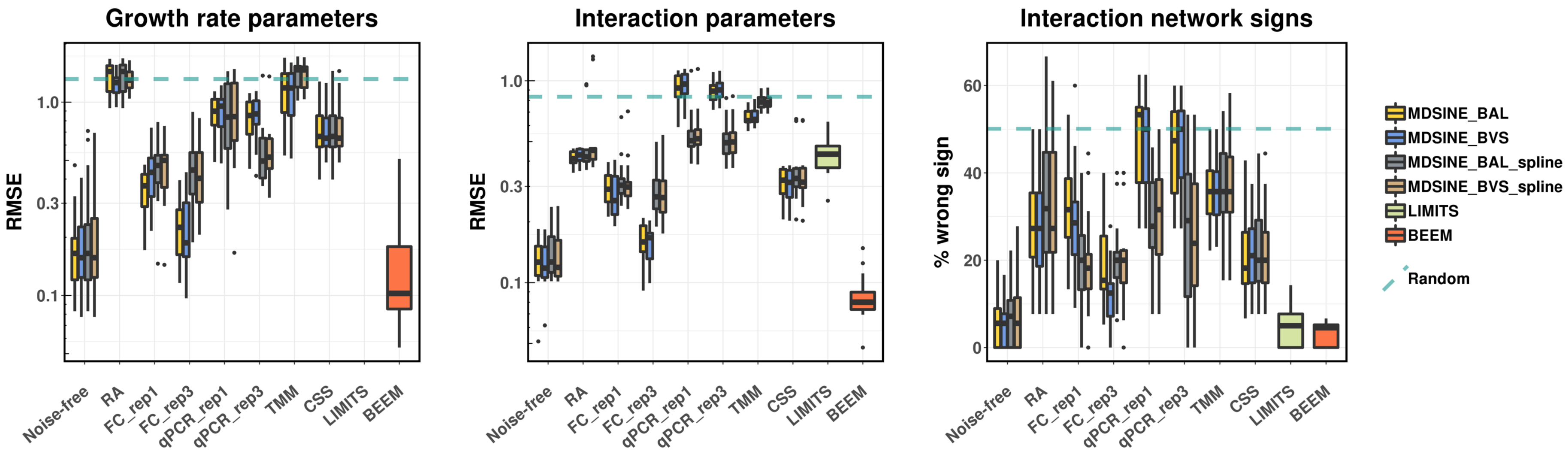

Supplementary Figure 2. The impact of noise on the performance of different gLVM parameter estimation algorithms is similarly captured with other evaluation metrics as well. The evaluation metrics were changed to root mean squared error (RMSE) and percentage of interaction signs that were wrongly predicted ("% wrong sign"), compared to relative error in **Supplementary Figure 1B**. Dashed horizontal lines represent the performance of randomly generated parameters from the simulation model. In general, scaling with noise-free biomass and using BEEM provided notably good performance, and among competing experimental (FC_rep1, FC_rep3, qPCR_rep1, qPCR_rep3) and computational approaches (RA – relative abundance, CSS – CSS normalization, TMM – TMM normalization, LIMITS), having three replicates from flow cytometry was the closest (FC_rep3). Note that LIMITS does not compute growth rate parameters.

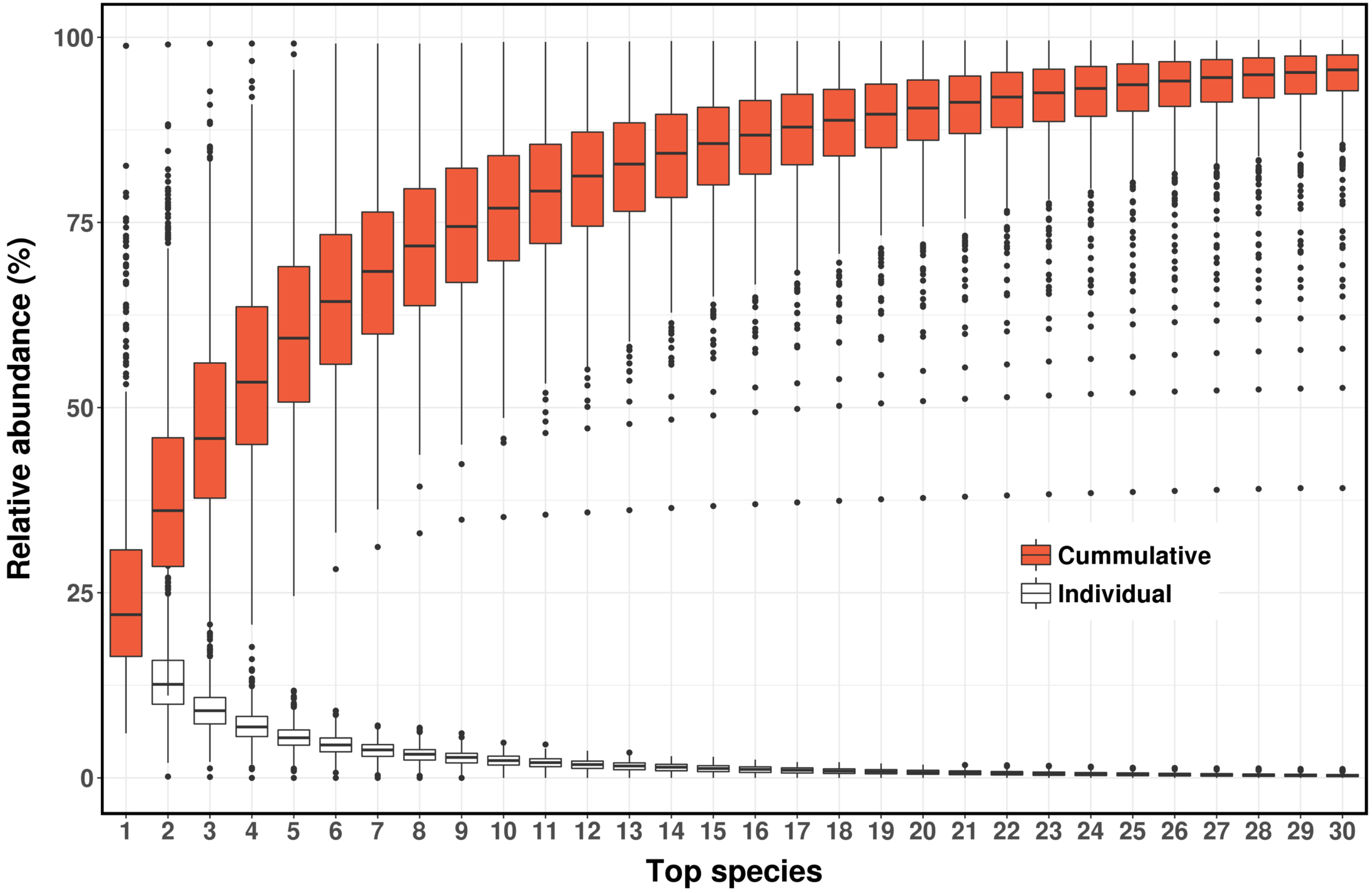

Supplementary Figure 3. Relative abundances observed for the most abundant species in 840 normal stool metagenomic samples from Pasolli *et al*. Filled boxplots show cumulative values.

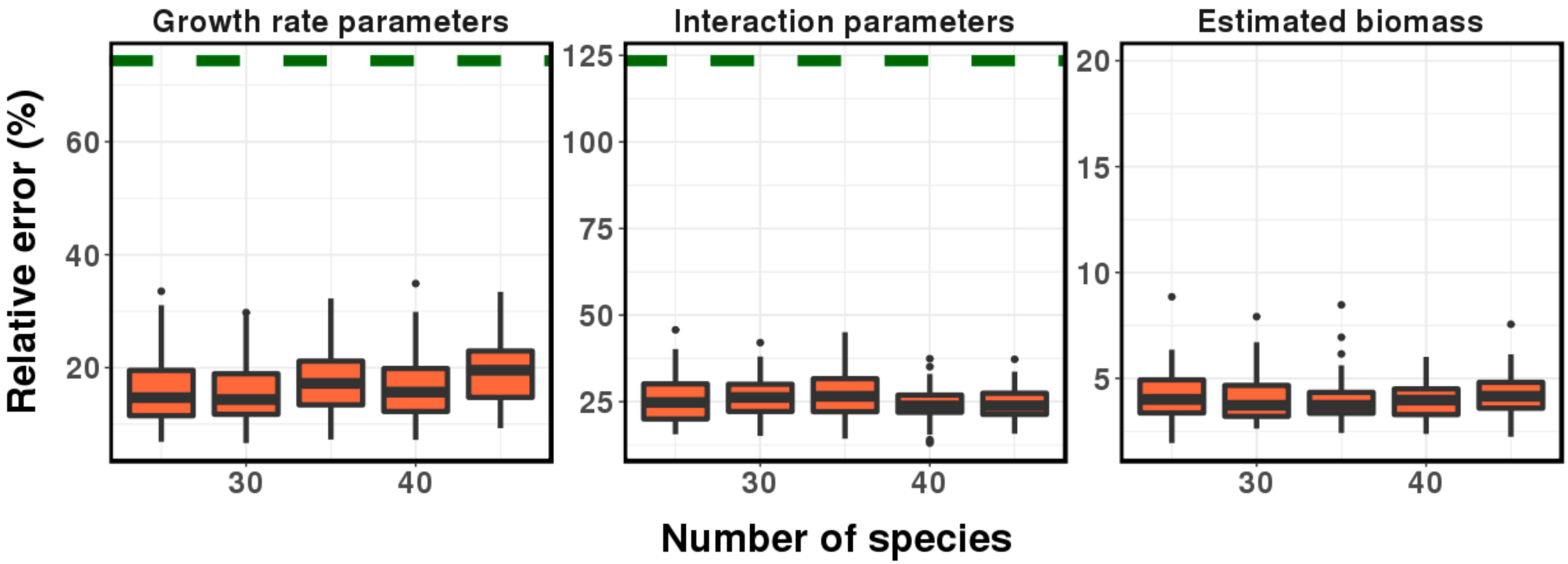

Supplementary Figure 4. Boxplots of relative error in BEEM estimated parameters from data with different number of species. Each box represents 30 independent simulations. For a community with p species, gLVM models have p(p+1) parameters (7p non-zero in the simulation). Given n microbiome profiles, we have at most n×p datapoints and therefore need n×p > p(p+1) or n > p+1. In practice, more data is needed and we simulated 2p replicates (20 timepoints each, to allow accurate spline-fitting), such that the number of replicates scales linearly with the number of species. Dashed horizontal lines represent the performance of randomly generated parameters from the simulation model.

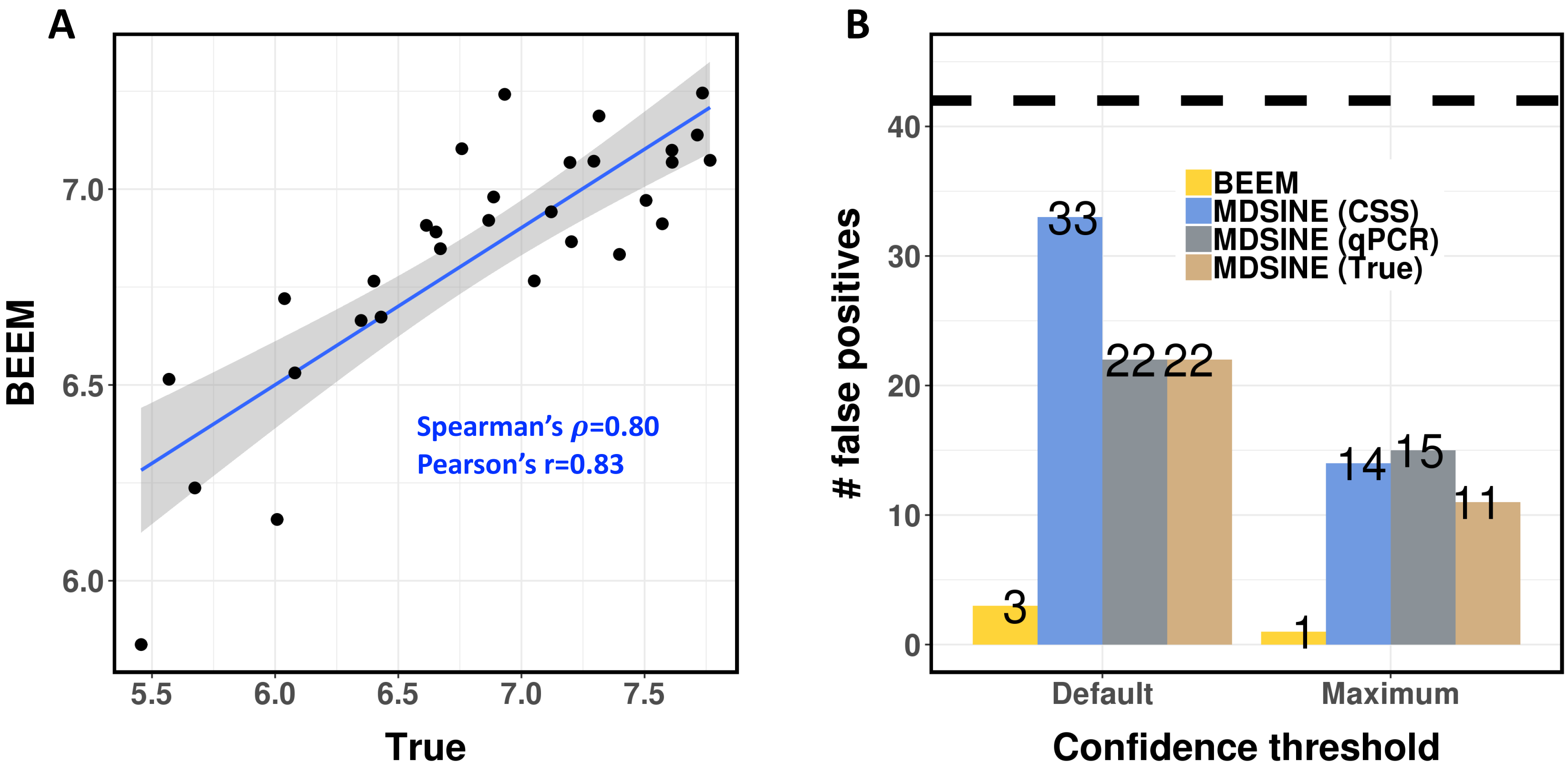

Supplementary Figure 5. BEEM effectively controls for false positive interactions in a synthetic community with no interactions (7 species, 3 replicates with 10 time points each). (A) Scatter plot with fitted linear regression line for BEEM estimated biomass and the true biomass (*p*-value<10^{-5}). (C) Bar plot for the number of false positive interactions detected using lenient (software recommended thresholds: ≥2 for BEEM and ≥10 for MDSINE) or strict (≥the maximum confidence value) confidence filter. Dashed line represents the total number of interaction parameters (42) to infer (CSS – CSS normalization, qPCR – simulated qPCR data with 3 replicates, True – true biomass).

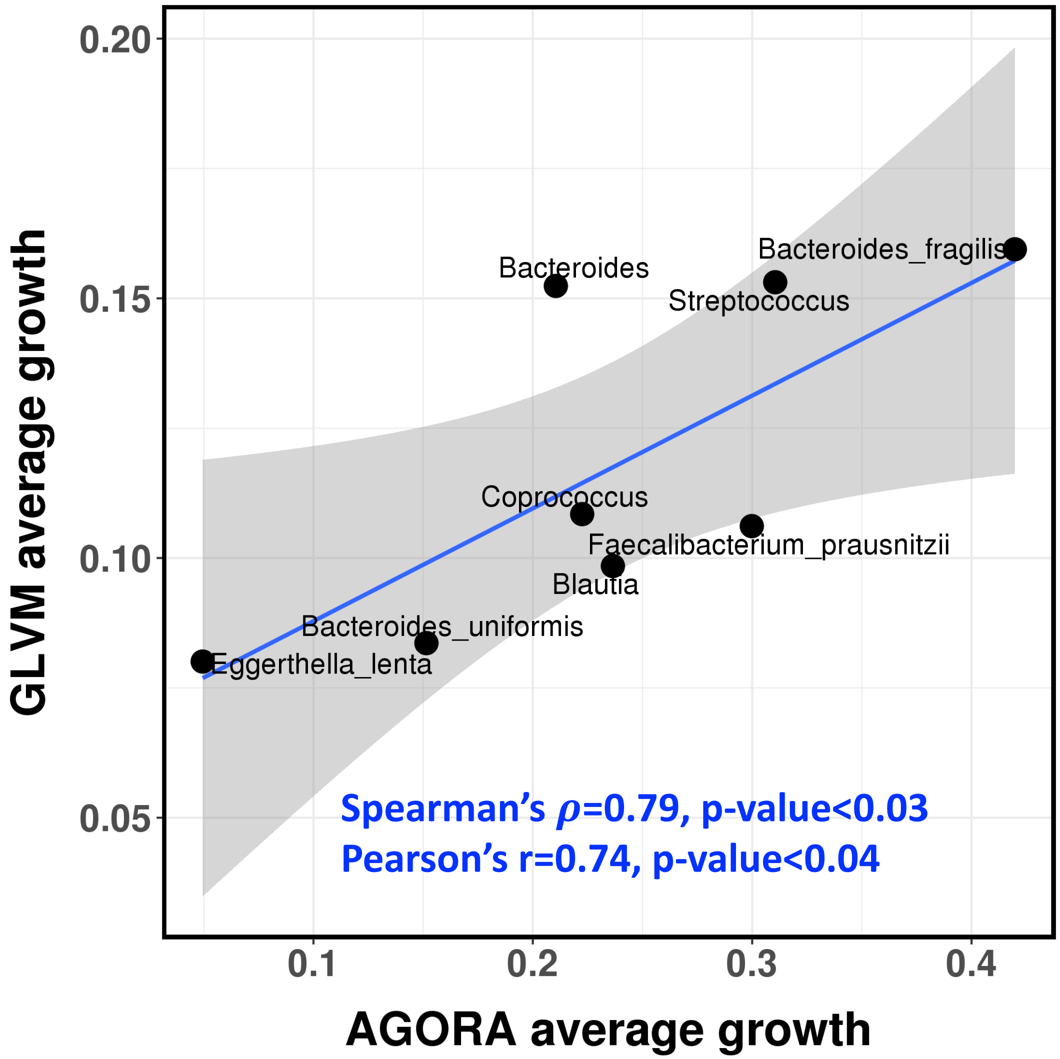

Supplementary Figure 6. Scatter plot for predicted growth rates from BEEM (average of ≥2 predictions) versus growth rates reported in the AGORA database (average across diet types under anaerobic conditions) based on genome-scale metabolic models.

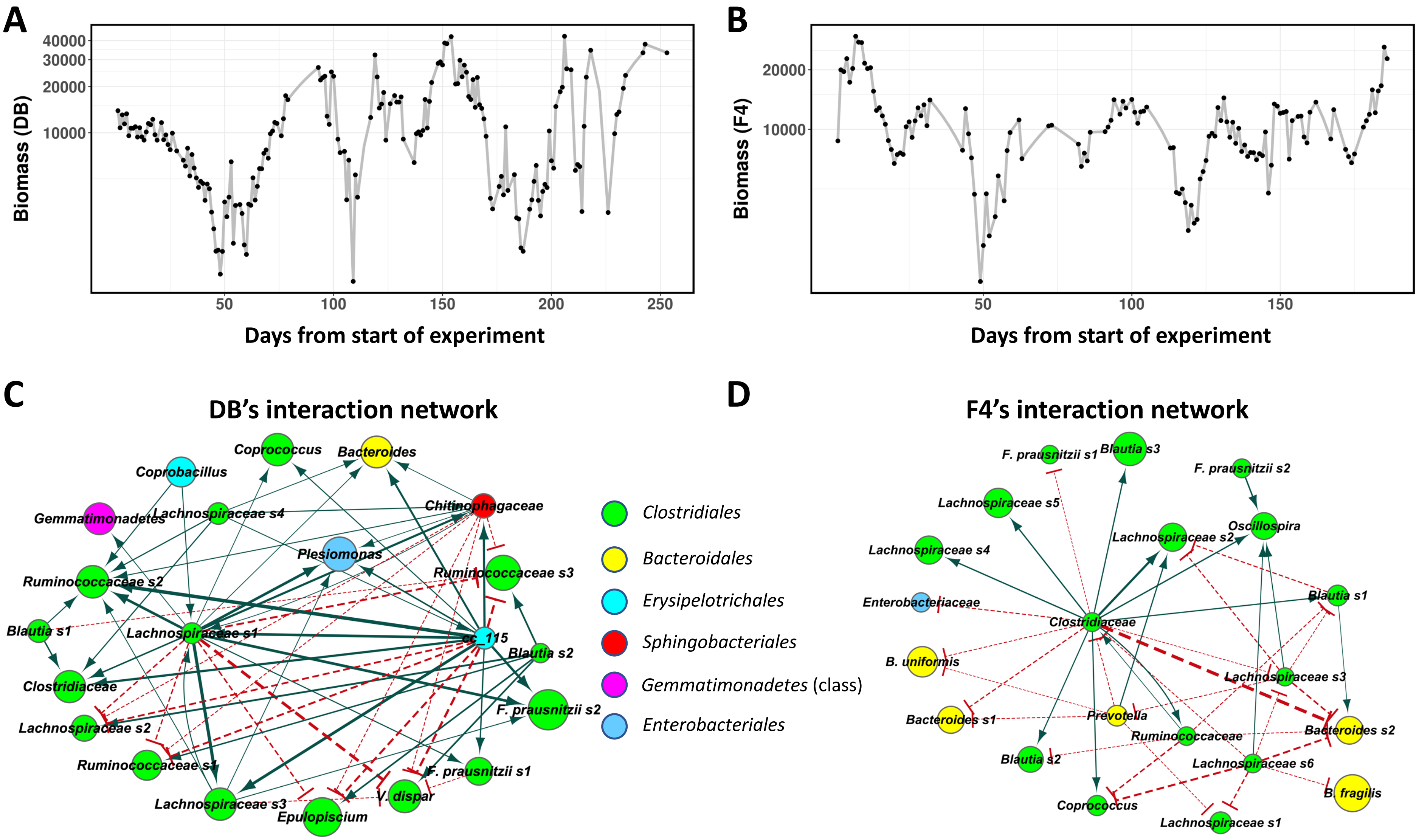

Supplementary Figure 7. BEEM estimated biomass (A and B; scaled to median of 10^4) and interaction networks (C and D) from the two shorter gut microbial longitudinal profiles from David *et al* and Caporaso *et al*. Dashed and solid edges represent positive and negative interactions, respectively, in the networks. Edge widths are proportional to the interaction strength and node sizes are proportional to log-transformed mean relative abundance of the corresponding species (OTU). Nodes are labeled with the most specific taxonomic annotation and colored according to order level of taxonomic annotation, where feasible.

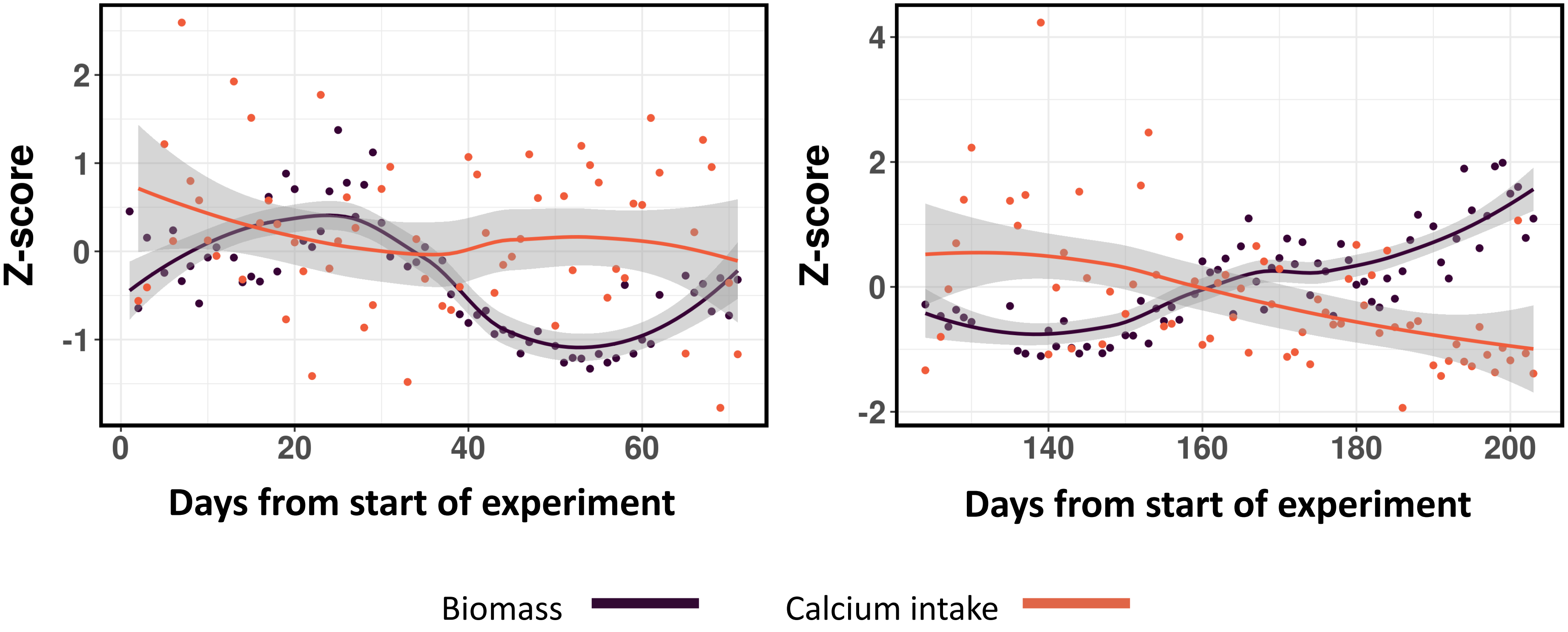

Supplementary Figure 8. Changes in calcium intake (z-score normalized) for the preceding day (orange) in relation to BEEM-estimated biomass (z-score normalized) for subject DA's gut microbiome (purple; only time points with calcium intake data are shown). Lines represent loess smoothers and shaded regions depict 95% confidence intervals. Overall, the two variables were found to be significantly correlated (Spearman's ρ=-0.40, FDR corrected q-value=1.7×10^{-5}; Pearson's r=-0.40, FDR corrected q-value=2.3×10^{-5}).

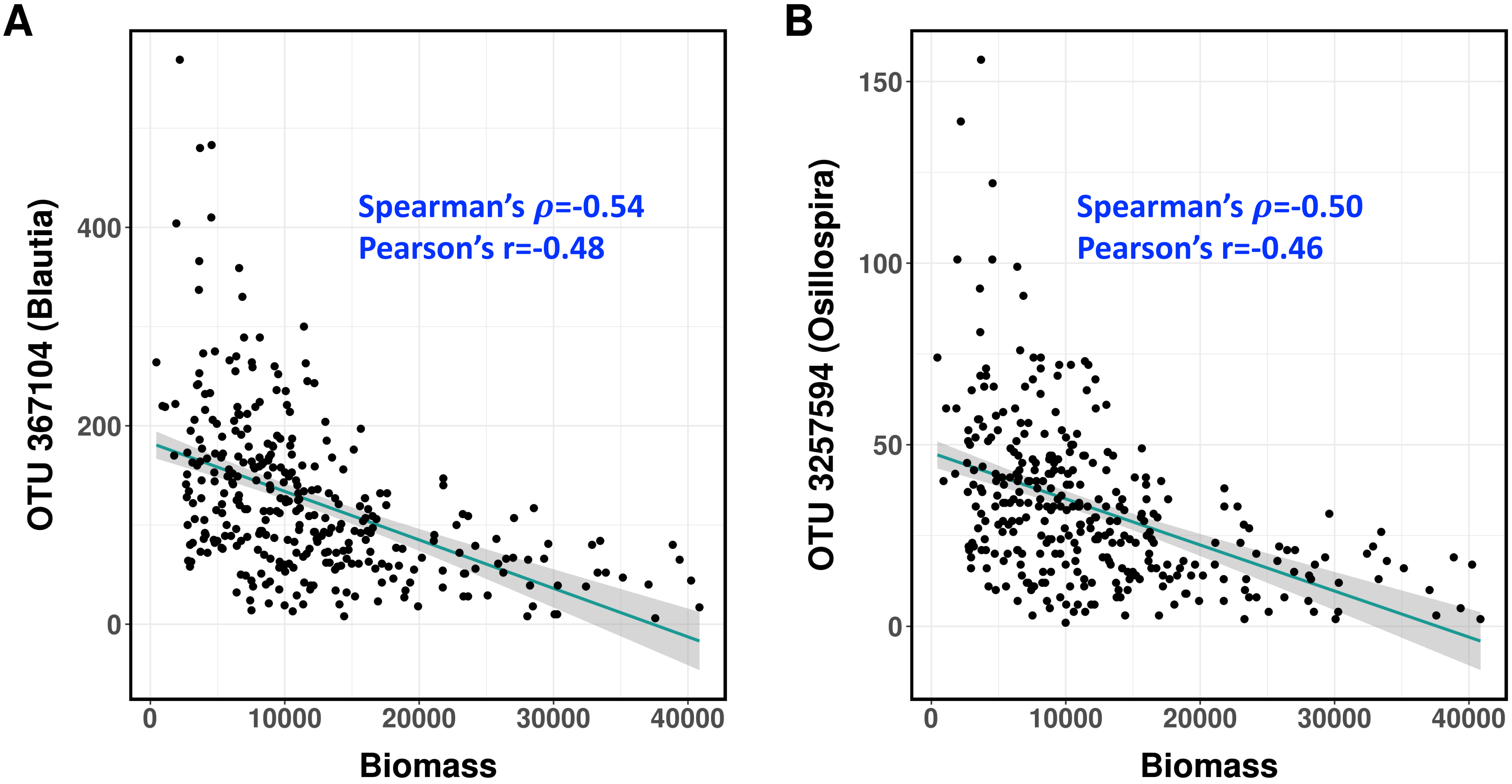

Supplementary Figure 9. Scatter plots with fitted linear regression lines between the two hub OTUs and the estimated biomass of M3's gut microbiome. All correlations are significant after FDR correction for multiple hypothesis testing (*q*-value$<10^{-16}$).

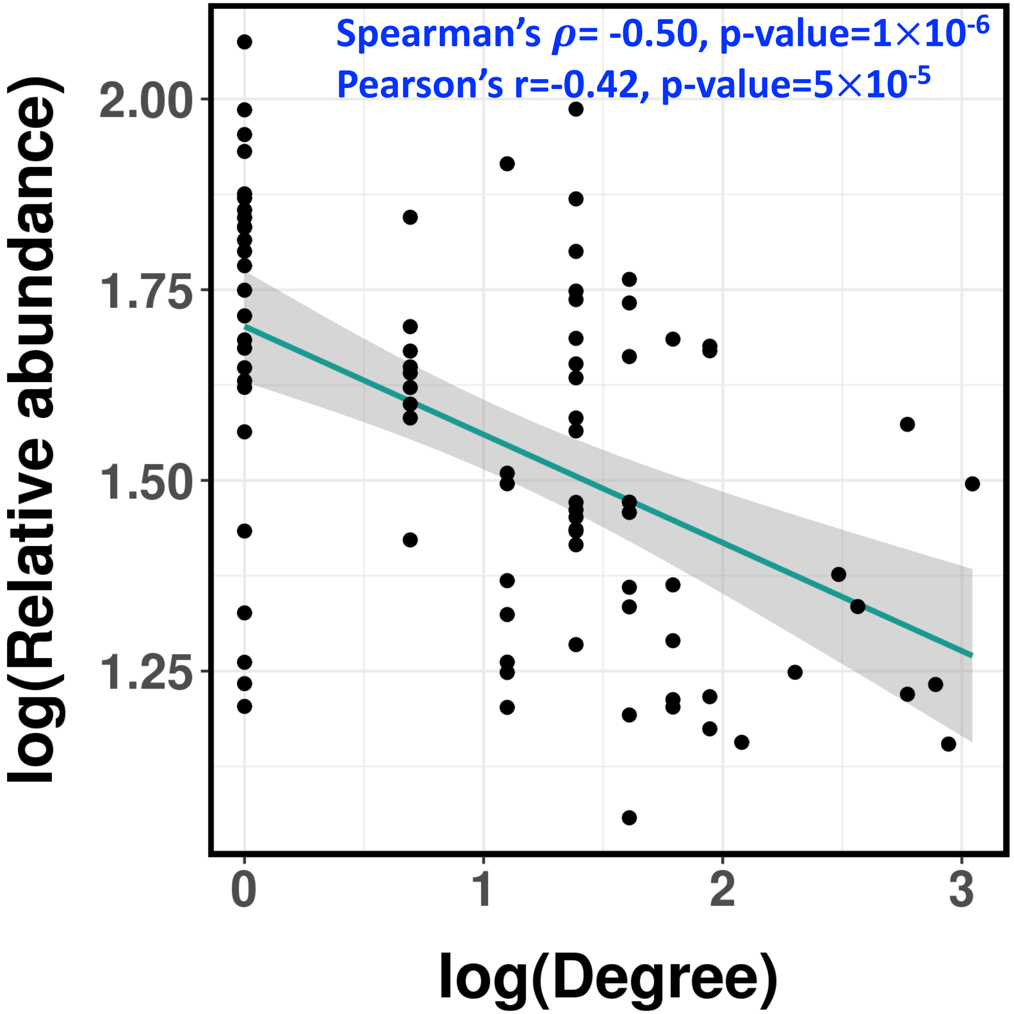

Supplementary Figure 10. Scatter plot with fitted linear regression line between the out- and in-degree of the OTU versus its mean relative abundance on log scale (based on networks for all 4 subjects).

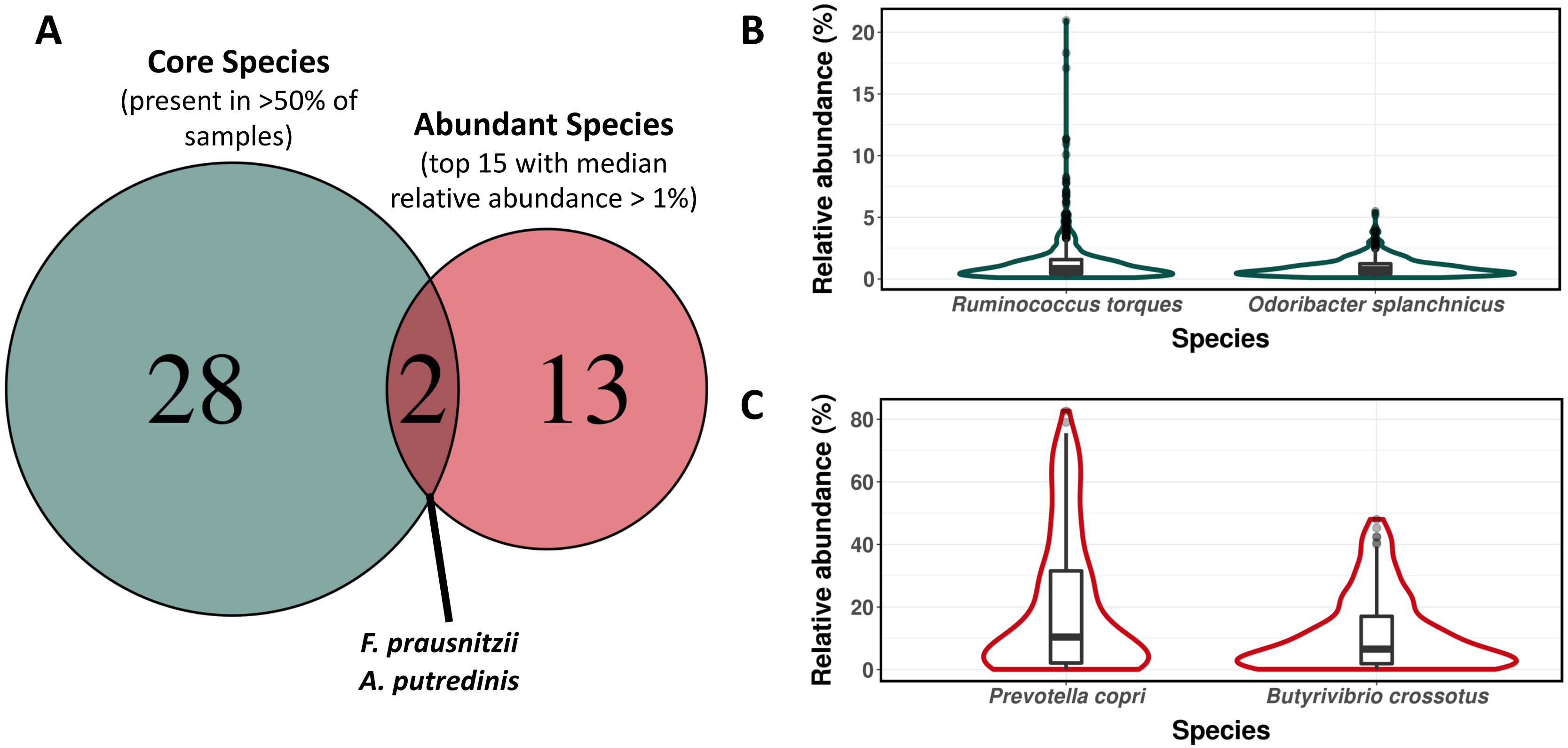

Supplementary Figure 11. (A) Venn diagram for core species (present in >50% of samples) and abundant species (top 15 with median relative abundance > 1%) in healthy human gut microbiomes from Pasolli *et al* (N=840). (B) Examples of core gut species with low relative abundances. *R. torques* and *O. splanchnicus* were present in 95% and 69% of samples but both of them rarely have relative abundance >1%. (C) Examples of more abundant species that are only found in a fraction of individuals. *P. copri* and *B. crossotus* are frequently present at abundances >5%, but are only present in 34% and 15% of all samples, respectively.
